# Stereo and LiDAR Loosely Coupled SLAM Constrained Ground Detection

**DOI:** 10.3390/s24216828

**Published:** 2024-10-24

**Authors:** Tian Sun, Lei Cheng, Ting Zhang, Xiaoping Yuan, Yanzheng Zhao, Yong Liu

**Affiliations:** 1 School of Mechanical Engineering, Shanghai Jiao Tong University, Shanghai 200240, China; jiaodat@sjtu.edu.cn (T.S.); yzh-zhao@sjtu.edu.cn (Y.Z.); 2 School of Computer Science and Engineering, Nanjing University of Science and Technology, Nanjing 210094, China; 18851792895@163.com; 3 School of Computer and Electronic Information, Nanjing Normal University, Nanjing 210023, China; zting135@nnu.edu.cn; 4 School of Information and Control Engineering, China University of Mining and Technology, Xuzhou 221116, China; xpyuankd@163.com

**Keywords:** pose optimization, simultaneous localization and mapping, point cloud, 3D reconstruction, sensor fusion

## Abstract

In many robotic applications, creating a map is crucial, and 3D maps provide a method for estimating the positions of other objects or obstacles. Most of the previous research processes 3D point clouds through projection-based or voxel-based models, but both approaches have certain limitations. This paper proposes a hybrid localization and mapping method using stereo vision and LiDAR. Unlike the traditional single-sensor systems, we construct a pose optimization model by matching ground information between LiDAR maps and visual images. We use stereo vision to extract ground information and fuse it with LiDAR tensor voting data to establish coplanarity constraints. Pose optimization is achieved through a graph-based optimization algorithm and a local window optimization method. The proposed method is evaluated using the KITTI dataset and compared against the ORB-SLAM3, F-LOAM, LOAM, and LeGO-LOAM methods. Additionally, we generate 3D point cloud maps for the corresponding sequences and high-definition point cloud maps of the streets in sequence 00. The experimental results demonstrate significant improvements in trajectory accuracy and robustness, enabling the construction of clear, dense 3D maps.

## 1. Introduction

Accurate localization is imperative for the effective operation of autonomous vehicles. The previous research has explored various sensor options for localization in complex environments. Although LiDAR is widely regarded as a reliable sensor for mapping and localization due to its precise range measurements, its high cost and inability to extract object colors pose challenges for widespread applications. Conversely, the GPS excels in signal-rich areas but falters in accurately localizing vehicles within urban and indoor environments. Cameras have emerged as a cost-effective alternative to LiDAR, boasting advantages such as affordability, compact size, and the ability to capture color information. However, monocular cameras suffer from a critical drawback known as scale uncertainty, leading to potential angular drift. To address this limitation, stereo camera systems have been proposed, effectively mitigating the issue of scale uncertainty. Nevertheless, their accuracy and robustness still lag behind those of LiDAR systems.

Different situations entail varying precision and system cost requirements [1]. Mapping requires high-precision sensors, and, in this study, sensitivity to cost is less critical since the equipment can be reused. In autonomous vehicle localization, as the number of vehicles increases, the processing load on the sensors also rises, often leading to errors in feature processing and reduced SLAM accuracy. To balance precision and cost, it is necessary to use high-precision yet cost-effective sensors. An effective approach to reducing costs while maintaining accuracy is to leverage the strengths of both cameras and LiDAR. Generally, there are two methods for fusing LiDAR and cameras [2]. The first method involves synthesizing RGB images from the LiDAR map [3,4]. This approach requires solving the relative extrinsic parameters of the camera and LiDAR, but the computational load for mapping images to the point cloud is substantial, making it unsuitable for localization systems with strong real-time performance requirements. The second method involves creating landmarks in the fused map [5,6], such as by identifying lane lines to aid visual localization. However, this method lacks generality and may not perform well on certain road types, such as rural roads. Therefore, the performance of the aforementioned methods is suboptimal in complex and dynamic environments.

**Figure 1:**
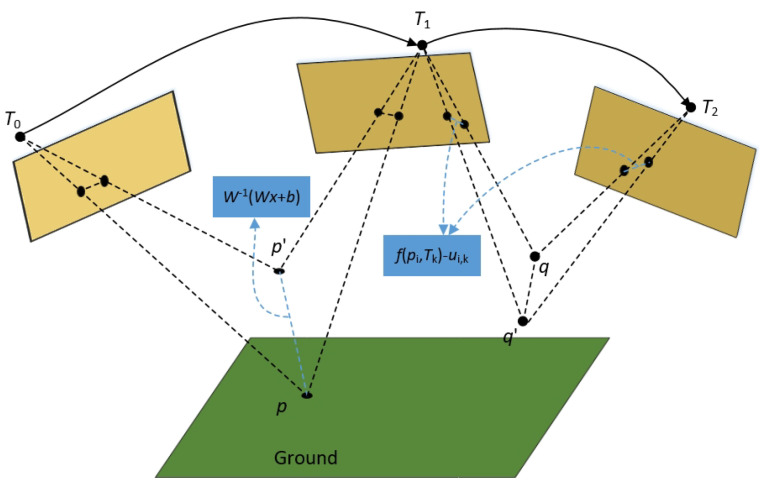
Pose optimization based on ground information. *T*, *p*, and *q* represent the transformation matrix, points on the plane, and points off the plane, respectively.

Calibration is required for multi-sensor fusion methods, and the accuracy of the calibration results can affect the accuracy of localization. Unlike directly fusing point clouds and images, we propose a method for localization using stereo cameras to avoid the need for sensor calibration. This method extracts geometric features from a LiDAR map previously generated by algorithms like G-LOAM [7] and then selects points with the same geometric properties in the visual image. These points satisfy both the bundle adjustment (BA) constraints and geometric constraints, as illustrated in Figure 1. Urban environments are primarily characterized by flat ground features. This work leverages our previous research on accurately identifying ground features [8] and the advantages of tensor voting [9] in spatial plane segmentation to design a tightly coupled trajectory estimation system. In summary, the contributions of this paper are outlined as follows:

(1) A 3D SLAM framework has been developed, which significantly reduces cumulative trajectory errors while enabling the construction of clear 3D reconstructions.
(2) A method that tightly couples stereo vision images and LiDAR point clouds by relying on ground information is proposed.
(3) A method for reconstructing 3D point clouds from spatial features using local bundle adjustment is employed.

The rest of this paper is structured as follows. In Section 2, we review related works concerning the evolution of SLAM and the challenges it encounters. Section 3 elucidates the proposed visual geometry and disparity calculation. The point cloud segmentation and extraction algorithm is presented in Section 4. The pose optimization is detailed in Section 5. The experimental results and future directions for this work are discussed in Section 5 and Section 6, respectively.

## 2. Related Work

In the past few decades, significant advancements have been made in both vision-based [10,11] and LiDAR-based [12] localization technologies. Methods based on cameras and LiDARs are frequently used for mobile robot localization. Vision sensors can detect the color features of the surrounding environment, while LiDARs provide precise distance information and exhibit strong robustness to varying lighting conditions.

Using laser radar cameras effectively for localization and mapping is a great choice, which can significantly enhance the applicability of algorithms. For instance, algorithms that fuse three types of sensors can effectively operate in scenes with poor lighting conditions or degraded structures. In recent years, there has been extensive literature on SLAM methods that integrate laser radar and cameras. Zhang et al. proposed DEMO [13], which utilizes depth values from laser point clouds to provide depth for some visual feature points. Compared to purely visual methods, DEMO achieves higher pose accuracy and provides higher-quality point cloud maps. Zhang et al. [14,15] further proposed the V-LOAM algorithm, which fuses multiple sensors in a unified optimization-based estimator framework to fully exploit the advantages of different sensors. V-LOAM can operate in highly dynamic scenes, demonstrating strong robustness and scene adaptability. V-LOAM is particularly prominent for its high precision and low drift in the renowned KITTI odometry evaluation dataset [16]. Graeter et al. proposed LIMO [17], which employs a novel approach to provide depth to image feature points using radar point clouds and assigns different weights to visual feature points based on semantic labels obtained from neural networks to better utilize sensor data.

Due to the relatively high cost of LiDAR-based methods and the inability of the traditional vision-based approaches to meet localization accuracy requirements, map-based visual localization has gradually emerged as a hot research topic. In [18], a monocular camera localization method utilizing a 3D prior map generated by LiDAR was proposed. The innovation lies in generating synthetic views from various poses using a GPU and subsequently computing the normalized mutual information (NMI) between these synthetic views. The pose with maximized NMI is identified as the desired pose. However, the generated map does not include height information. In a different approach, ref. [19] presented a monocular-vision-based urban localization technique focusing on road lane identification using a random forest-based edge detector. The Chamfer distance is computed between the detected edges and projected road marking points in the image space. Non-linear optimization, incorporating epipolar geometry constraints and odometry, is then employed to estimate the six-degrees-of-freedom camera pose. The authors in [20] utilized Semi-Global Block Matching (SGBM) to estimate the disparity and recover depth from stereo images. The depth from the stereo camera is matched with a prior LiDAR map, and a complete six-degrees-of-freedom camera pose is estimated by minimizing the depth residual. Some scholars have explored cloud robotic systems [21,22], with plans to apply the developed methods to future cloud robot work.

In the field of visual-LiDAR SLAM, concurrent strategies with multiple sensors, LiDAR-assisted visual SLAM, camera-supported LiDAR SLAM, and tightly coupled fusion methods have been explored [23]. LIMO SLAM [17] models local planes using LiDAR depth information and extracts depth features from camera images. Shin et al. [24] integrated visual and sparse LiDAR depth data for SLAM, employing optimized sliding window techniques and incorporating loop closure functionality, achieving significant performance across various large-scale datasets. LIC-fusion (LiDAR-Inertial-Camera) [25] ensures good localization accuracy and robustness by integrating LiDAR features, camera features, and high-frequency IMU data with online multi-sensor calibration capabilities. Chou et al. [26] proposed a tightly integrated visual-LiDAR SLAM, termed TVL-SLAM, with separate frontends for LiDAR and camera, fused in the backend for pose optimization with LiDAR loop closure. TVL-SLAM outperforms single visual and LiDAR SLAM implementations.

R2 LIVE [27] combines an error-state Kalman filter with factor-graph optimization to estimate trajectories, integrating LiDAR, IMU, and visual measurements. Its successor, R3 LIVE [28], took a further step by integrating a colored 3D map, a combination of VIO and LIO subsystems, and online photometric calibration, ensuring robustness against camera failures and LiDAR degradation scenarios, even on small onboard platforms, showcasing remarkable performance. Super Odometry [29], a brainchild of Zhao et al., emerged as an IMU-centric robust state estimation pipeline, demonstrating remarkable efficacy, particularly in challenging environments with degraded perception.

AdVLO [30] uses an attention mechanism to select key regions, fusing visual and LiDAR features for improved odometry estimation. Nowakowski [31] proposed combining YOLO and laser scanning to enhance target detection and precise distance estimation on unmanned platforms. Further, 3D cameras and LiDAR help small robots to navigate autonomously and weed in orchards, overcoming canopy navigation challenges [32]. These methods have been validated on the KITTI dataset and in some orchard scenarios using semantic or depth map fusion techniques. This paper focuses more on research aimed at improving SLAM accuracy in urban environments.

Integrating multiple sensor measurements in a sensible manner often leads to performance improvements, but algorithm designers should also be mindful of additional considerations. To fuse data from various sensors effectively, the calibration of the extrinsic parameters between different sensors is necessary, which includes the six-degrees-of-freedom (six-DoFs) relative pose transformation and time offset. Achieving accurate extrinsic parameters between sensors of different modalities typically requires complex offline calibration processes. Over prolonged usage, mechanical deformations may cause variations in the extrinsic parameters between sensors. When precise calibration of extrinsic parameters cannot be obtained, online calibration within the estimator can be conducted to mitigate the effects of poor extrinsic parameters. However, online calibration of extrinsic parameters is susceptible to degradation motion effects. For example, pure translational motion or single-axis rotational motion in visual–inertial systems can render the translation component of camera and IMU extrinsic parameters unobservable [33].

In conclusion, the landscape of visual-LiDAR SLAM is rapidly evolving, propelled by innovations in sensor fusion, optimization techniques, and robust state estimation pipelines. From the integration of LiDAR depth data with visual SLAM frameworks to the advancement of tightly coupled sensor fusion methods, researchers are pushing the boundaries of precision, robustness, and real-time performance. As the field progresses, the seamless integration of multimodal sensor data continues to unlock new possibilities, offering enhanced navigation solutions across a diverse range of environments and scenarios. The ongoing pursuit of Super Odometry and similar breakthroughs underscores the quest for ever-improving performance, particularly in challenging perceptual conditions, signifying an exciting era of exploration and discovery in visual-LiDAR SLAM research.

## 3. Stereo Matching and Disparity Calculation

### 3.1. Stereo Geometry

**Figure 2:**
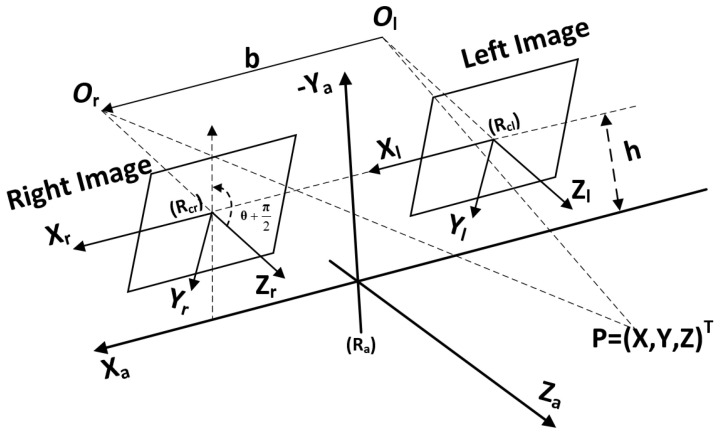
The stereo sensor model and the coordinate systems used [34].

A parallel stereo rig is set up as described in [34,35]. As depicted in Figure 2, the world coordinates of a 3D point P(X,Y,Z) are projected onto a 2D point p(u,v) in the image. $\left(u_{0},v_{0}\right)$ represents the optical center in the projected image coordinate. The coordinates (u,v) can be expressed as follows:

(1) $$\begin{array}{cc} u & =u_{0}+\frac{fX-\varepsilon_{i}\frac{b}{2}f}{(Y+h)\sin\theta +Z\cos\theta }\\ v & =v_{0}-f\tan\theta +\frac{fb}{\cos\theta \left[(Y+h)\cos\theta +Z\cos\theta \right]}. \end{array}$$

where *i* represents either the left or right camera. $\varepsilon_{l}$ = −1 and $\varepsilon_{r}$ = 1. The camera’s pitch angle and height are denoted as θ and *h*, respectively. The stereo baseline and the measured local length are represented by *b* and *f*, respectively.

Subsequently, the disparity $\Delta =u_{l}-u_{r}$ is calculated as

(2) $$\begin{array}{c} \Delta =\frac{fb}{(Y+h)\sin\theta +Z\cos\theta }. \end{array}$$

A map that includes the disparity value for each pixel is referred to as a disparity map.

(3) $$\begin{array}{c} \Delta =\frac{b(v\cos\theta -v_{0}\cos\theta +f\sin\theta )}{\left(Y+h\right)}. \end{array}$$

### 3.2. Positive Obstacle Removal

The fast and effective obstacle removal scheme is based on the concept of u-disparity. Peaks in the u-disparity are identified using a threshold denoted as $O_{b}$. Any point with a u-disparity value greater than $O_{b}$ is classified as an obstacle point. Subsequently, a new disparity map is generated from the original one after the identified positive obstacles have been removed. The primary objective of this pre-processing step is to aid in extracting the ground profile rather than attempting to identify all obstacle pixels in the disparity map.

**Figure 3:**
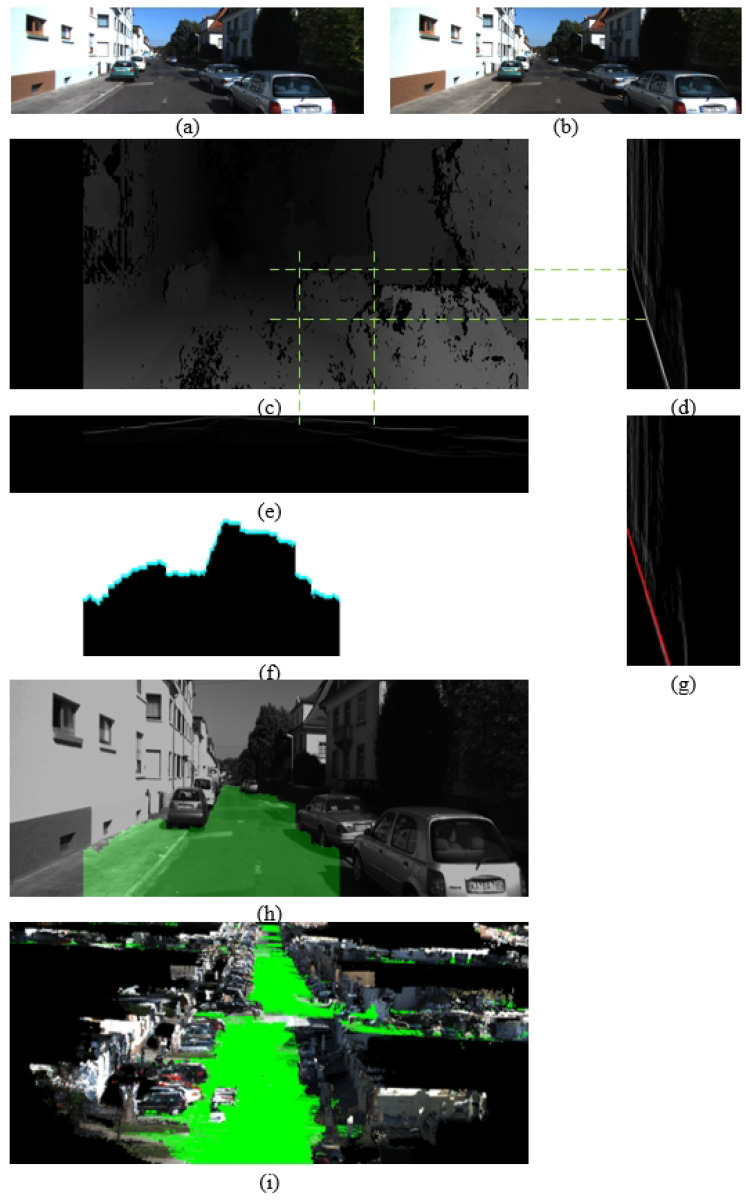
Region of interest extraction. (**a**) Left image. (**b**) Right image. (**c**) Disparity image. (**d**) v-disparity. (**e**) u-disparity. (**d**,**e**) are derived from (**c**). (**f**) Large obstacles removed by removing peak values from (**e**). (**g**) v-disparity based on (**f**), and red line is disparity profile of ground plane. (**h**) Detected ground plane and region of interest (RoI); RoI in red box. (**i**) City 3D reconstruction; green represents ground.

As depicted in Figure 3, elements such as the sky, trees, roadside features, and pedestrians are effectively eliminated through this initial positive obstacle removal process. This refinement leads to a more accurate definition of the region of interest (ROI). Consequently, it results in enhanced detection accuracy, as further discussed in the subsequent section.

### 3.3. Ground Detection

We use a stereo camera for plane detection, and an example of a disparity image is shown in Figure 3.

We referred to the method outlined in [34] and utilized the u-disparity and v-disparity of the disparity map for ground estimation. Specifically, the u-disparity is generated along the image rows, while the v-disparity is generated by computing the disparity histogram along the rectified image columns. Regions with peak disparitites are identified as large obstacles. To estimate the ground surface, we employed non-parametric road detection [35], a fast and efficient method suitable for non-planar ground surfaces.

In this method, the u-disparity is first calculated from the disparity map, and peak values in the u-disparity image (considered large obstacles) are removed. Points with a u-disparity greater than $O_{b}$ are treated as peaks. The value of $O_{b}$ is calculated using the formula $O_{b}=\left(H_{o}\times d\right)/b$ (where *d* represents the disparity value), with $H_{o}$ being the minimum height required for an obstacle. To expedite the process, we store the calculated thresholds for each disparity as a lookup table.

Removing large obstacles before generating the v-disparity enables more accurate ground detection. Therefore, while traversing the v-disparity row by row, we have already removed the disparity values considered as large obstacles. Through this process, we can estimate the disparity values for each row and for all pixels on the ground. The overall process is illustrated in Figure 3.

## 4. Groundplane Detection in LiDAR

In the pursuit of creating high-precision maps using LiDAR and GPS data, it becomes essential to extract relevant geometric information from the map. This information serves as crucial constraints for visual positioning. Through our observations, we have verified that plane features hold significant value, especially in urban environments. The extraction of these plane features from point cloud maps is a relatively straightforward process.

In our proposed method, we utilize the homogeneous coordinates of points in 2D and 3D as presented below.

(4) $$\begin{array}{c} p=\left[\begin{array}{c} u\\ v\\ 1 \end{array}\right]\mathrm{and}\;\;P=\left[\begin{array}{c} x\\ y\\ z\\ 1 \end{array}\right] \end{array}$$

The camera’s pose is depicted through an SE(3) transformation.

(5) $$\begin{array}{c} T=\left[\begin{array}{cc} R & t\\ 0 & 1 \end{array}\right]\mathrm{where}\;\;T\in \mathrm{SE}\left(3\right), \end{array}$$

which is related to its corresponding se(3) Lie algebra $\xi^{\wedge }$ by exponential map

(6) $$\begin{array}{c} T\left(\xi \right)=exp\left(\xi^{\wedge }\right) \end{array}$$

The wedge operator ∧ turns $\xi \in R^{6}$ into a member of the se(3) Lie algebra. For tracking and localization, camera pose *T* is iteratively updated as

(7) $$\begin{array}{c} T\leftarrow T(\xi )\cdot T \end{array}$$

where the increment *T*(ξ) is applied by using left multiplication convention.

The processing of the point cloud map can be broken down into two distinct steps. Initially, we employ the tensor voting algorithm, as outlined in [9,36], to compute the normal vectors for each point within the point cloud. Subsequently, in the second step, we leverage the k-means algorithm to group points with similar normals. Based on the outcomes of this clustering process, we calculate the equations defining the planes.

Tensor voting has proven to be an effective technique for determining surface normals. Its fundamental concept revolves around extracting implicit geometric characteristics from extensive and scattered point cloud data by facilitating the transfer of tensors between adjacent points. As the distance between points increases, the influence of points within the voting field gradually diminishes. Adhering to this principle, we establish the tensor kernel as follows:

(8) $$\begin{array}{c} k\left(d,\sigma \right)=e^{-\frac{d}{\sigma^{2}}} \end{array}$$

The computation involves the distance metric $d={\left(x_{i}-x\right)}^{2}+{\left(y_{i}-y\right)}^{2}$, where (x,y) denotes the coordinates of the point receiving the vote and $\left(x_{i},y_{i}\right)$ represents the coordinates of the voting point. The parameter σ signifies the kernel size of the sparse voting field. Representing the input point *P* is achieved through a second-order symmetric semi-positive definite tensor, denoted as *S*. This tensor can be expressed as a positive semidefinite matrix $S_{3\times 3}$ due to the equivalence between tensors and matrices. Furthermore, it can be decomposed into three distinct components, taking the following forms:

(9) $$\begin{array}{ccc} S & =\lambda_{1}{\hat{e}}_{1}{\hat{e}}_{1}^{T}+\lambda_{2}{\hat{e}}_{2}{\hat{e}}_{2}^{T}+\lambda_{3}{\hat{e}}_{3}{\hat{e}}_{3}^{T}\\ \; & =\left(\lambda_{1}-\lambda_{2}\right){\hat{e}}_{1}{\hat{e}}_{1}^{T}+ & \left(stick\;\;component\right)\\ \; & \;\;\;\;\;\;\;\;\left(\lambda_{2}-\lambda_{3}\right)\left({\hat{e}}_{1}{\hat{e}}_{1}^{T}+{\hat{e}}_{2}{\hat{e}}_{2}^{T}\right)+ & \left(plate\;\;component\right)\\ \; & \;\;\;\;\;\;\;\;\lambda_{3}\left({\hat{e}}_{1}{\hat{e}}_{1}^{T}+{\hat{e}}_{2}{\hat{e}}_{2}^{T}+{\hat{e}}_{3}{\hat{e}}_{3}^{T}\right) & \left(ball\;\;component\right) \end{array}$$

Following the tensor voting framework, we can obtain the normal vectors for each point. According to Equation (2), we can identify the points on the ground that conform to the following characteristics:

(10) $$\begin{array}{c} P_{t}=\left(\lambda_{2}-\lambda_{3}\right)\left({\hat{e}}_{1}{\hat{e}}_{1}^{T}+{\hat{e}}_{2}{\hat{e}}_{2}^{T}\right) \end{array}$$

## 5. Pose Optimization

Visual points can be classified into two categories: points *p* lying on the ground and points *q* not on the ground. In the LiDAR coordinate system, the distance from a planar point *p* to a spatial point $p^{\prime }$ is ${\left|n\right|}_{2}^{-1}\left(n^{T}p+b\right)$. In the visual localization framework, point *q* only needs to minimize the reprojection error $\left(f\left(q_{i},T_{k}\right)-u_{i,k}\right)$ (where *f* is the mapping function from 3D to 2D), while point *P* must minimize both the reprojection error and the coplanarity error.

Traditional visual localization consists of three main stages. Firstly, the pose between two frames is calculated by matching features between them. This stage involves feature extraction and matching, typically using local invariant features such as ORB, SIFT, or SURF to describe key points in the images. By comparing descriptors, correspondences between frames are found, enabling the computation of their relative pose. Subsequently, depth information is reconstructed through triangulation. This step utilizes the camera projection relationship from two viewpoints. With known camera parameters and matched feature points, depth values are computed to build a 3D point cloud of the scene. Finally, bundle adjustment is performed based on the map points and corresponding frames. Bundle adjustment optimizes camera poses and 3D point cloud positions to minimize reprojection error, enhancing the accuracy and consistency of the entire system.

In this paper, our approach introduces projection constraints for ground points in addition to bundle adjustment during optimization. Traditional bundle adjustment methods consider only the reprojection error of feature points, neglecting the coplanarity constraints of ground points, which can lead to decreased accuracy and robustness of localization in dynamic environments or scenes rich in planar ground information. To address this issue, we incorporate additional projection constraints for ground points during the optimization process, leveraging ground plane information to enhance localization accuracy.

We employ an incremental sliding window method to connect observed points with the current point in each incoming frame. In this process, the target points within the frame are optimized concurrently with the observed images to determine a maximum six-degrees-of-freedom (six-DOFs) pose. This approach enables continuous and precise tracking of the camera’s movement, enhancing the overall robustness of the localization process.

We represent the Lie algebra corresponding to the rotation matrix R and translation vector *t* as $\xi^{\wedge }$. The Lie algebra provides a mathematical framework for representing the infinitesimal rotations and translations, enabling efficient optimization in the parameter space. This compact representation simplifies the process of updating the camera poses during the optimization. Here, p represents the observed map point, which is a 3D feature point identified and tracked across multiple image frames. These map points are critical for determining the geometric structure of the scene. The provided formula represents the error resulting from the observation of the *k*-th point in the *j*-th frame. This error is the difference between the actual observed position of the point in the image and its reprojected position based on the current estimates of the camera pose and the 3D point position.

The cost function, which we aim to minimize during bundle adjustment, is expressed as follows:

(11) $$\begin{array}{c} E_{ba}=\left|\right|{\left(u_{i}-Kexp\left(\xi^{\wedge }\right)p_{k}\right)}^{T}Q_{k,j}^{-1}(u_{i}-Kexp\left(\xi^{\wedge }\right)p_{k}){\left|\right|}_{2}. \end{array}$$

where $u_{i}$ represents the observed 2D pixel coordinates of point *i*. *K* is the camera intrinsic matrix, used to map 3D points from the camera coordinate system to the 2D image plane. Denotes the pose transformation representing camera motion, expressed using the exponential map from Lie algebra, where ξ represents the motion parameters.

For the points located on the plane, we introduce plane constraints as additional constraints using the sliding window algorithm. In the first phase, BA is applied to these points. As more plane information is introduced, the number of plane points gradually increases, and corresponding constraints are introduced, leading to more accurate pose estimation. The ground equation can be represented as Wx+b=0, and its projection cost function is defined as follows:

(12) $$\begin{array}{c} E_{p}=\|{\left(\frac{1}{{\left|n\right|}_{2}}\left(n^{T}T_{i}p_{j}+b\right)\right)}^{T}R_{j}^{-1}\left(\frac{1}{{\left|n\right|}_{2}}\left(n^{T}T_{i}p_{j}+b\right)\right){\|}_{2}^{2}. \end{array}$$

where *n* represents a weight used to describe the normal vector of the plane and *b* is a bias vector used to adjust the position of the plane. They are constrained by the vehicle pose $T_{i}$.

**Figure 4:**
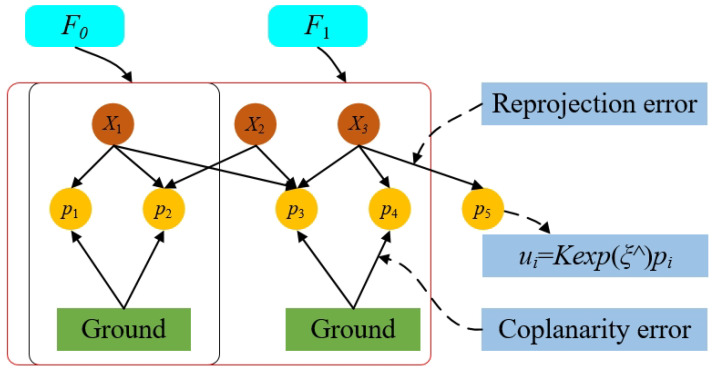
Graph-structure optimization. *P* represents the nodes of visual points, and *X* represents the pose of the frame. “Ground” denotes the ground information extracted from the 3D reconstruction.

We adopted a commonly used graph optimization method to address the optimization problem in visual localization, which is based on keyframes. We constructed a graph optimization model centered on the optimization problem, as shown in Figure 4, where each edge in the graph represents different constraints. The factor graph formulation is highly intuitive and has the advantage of enabling efficient implementations of batch and incremental solvers. Our optimization system includes both BA constraints and plane constraints.

In our model, we denote *p* as the node representing visual points, *X* as the node representing the frame’s pose, and $p_{g}$ as the node representing points on the plane extracted from the ground map. We also define the error function $r_{k}\left(p_{k},T\right)$ that quantifies the discrepancy between $p_{k}$ and the observed point, and we employ an edge to represent this relationship. Similarly, $r_{g}\left(p_{k},p_{g}\right)$ is defined as the cost function that projects a point onto the plane, analogous to $r_{k}\left(p_{k},T\right)$, and we also represent this relationship using an edge. We can utilize Equation (13) to formulate the optimization problem:

(13) $$\begin{array}{c} E\left(p\right)=\sum_{k=1}^{K}\sum_{j\in E(k)}r_{g}\left(p_{k},p_{g}\right)Q_{k,j}^{-1}r_{l}\left(p_{k},p_{g}\right)\\ +\sum_{p\in \pi_{i}}r_{g}{\left(p_{k},p_{g}\right)}^{T}R_{j}^{-1}r_{l}\left(p_{k},p_{g}\right) \end{array}$$

The Levenberg–Marquardt scheme is used to optimize the function, and the residual around the current state is linearized.

(14) $$\begin{array}{c} r\left(p⊕\delta s\right)=r\left(s\right)+J_{s}\delta s. \end{array}$$

In the function

(15) $$\begin{array}{c} J_{p}=\frac{dr(p⊕\delta p)}{2}{|}_{\delta p=0}. \end{array}$$

The error function E(s) is approximated with a quadratic function for the current state *s*:

(16) $$\begin{array}{cc} E(p⊕\delta p) & =E_{p}+\delta p^{T}b_{p}+\frac{1}{2}\delta p^{T}H_{s}\delta b_{p}\\ b_{p} & =J_{p}^{T}Wr\left(p\right)\\ H_{p} & =J_{p}^{T}WJ\left(p\right) \end{array}$$

where $b_{p}$ is the Jcaobian matrix and $H_{p}$ is the Hessian matrix. This function updated the state p←p⊕δp by minimizing $\delta p=-H^{-1}b_{p}$. We repeat this update and linearization process until convergence.

## 6. Experimental Results

This section provides a summary of the results obtained using our proposed method. Our goal is to conduct a comparative analysis of the current state-of-the-art systems, with a particular focus on accuracy. This article uses the left disparity image as the ground for image detection while mapping the LiDAR point cloud onto the left image. We conducted experiments to evaluate the performance of our proposed approach using the training dataset from the publicly available KITTI [37] benchmark. The dataset comprises sequences 00–10, each accompanied by ground truth trajectories. Our analysis focused on assessing the accuracy of our method. The experiments were conducted on a computer equipped with an Intel Core i5-12400F @ 4.2GHz processor and 32GB of memory, running a 64-bit Linux operating system.

### 6.1. Comparison with Others

**Figure 5:**
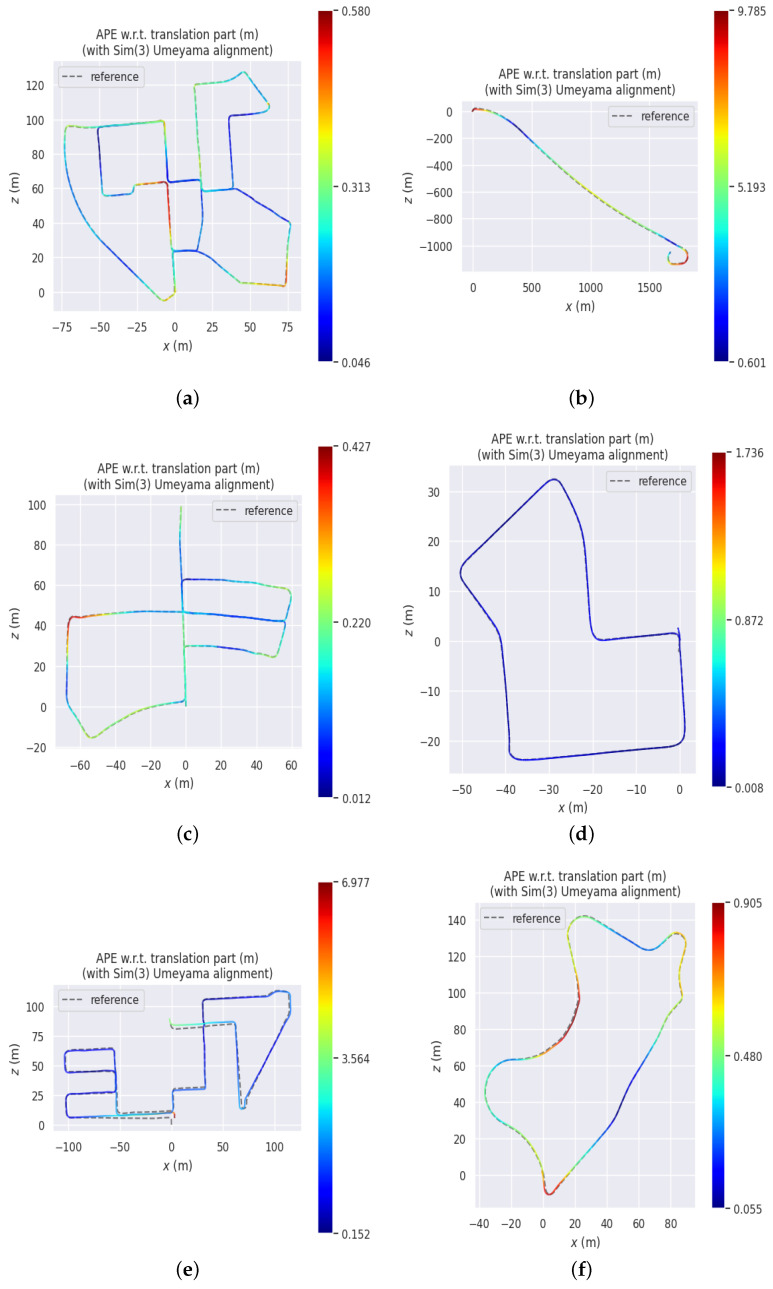
Trajectory estimates in the KITTI dataset. (**a**) 00. (**b**) 01. (**c**) 05. (**d**) 07. (**e**) 08. (**f**) 09.

**Figure 6:**
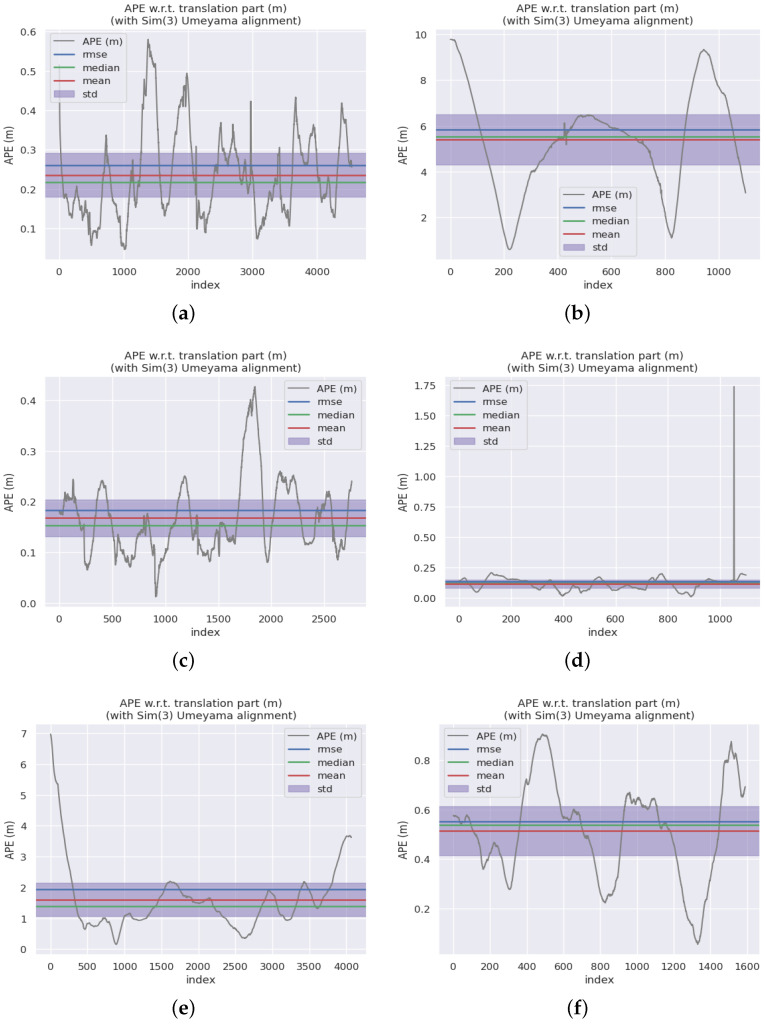
High-definition display of point clouds for some streets in the 00 sequence. (**a**) 00. (**b**) 01. (**c**) 05. (**d**) 07. (**e**) 08. (**f**) 09.

This paper compares the ORB-SLAM3 [38] stereo module, F-LOAM [39], LOAM [40], and LeGO-LOAM [41], as shown in Table 1. For fairness, we referenced [42] to provide the trajectory estimation of F-LOAM, LOAM, and LeGO-LOAM on sequences 00–10 of the KITTI dataset. Compared to these methods, our approach has achieved an average improvement of at least 63.64% in RMSE and 65.51% in standard deviation when evaluated using the ATE method. When evaluated using the RPE method, our approach has achieved an average improvement of at least 52.15% in RMSE and 59.08% in standard deviation. Figure 5 illustrates the trajectories of 11 representative sequences. The gray dashed line in the figure represents the ground truth of the dataset, while the colored solid line indicates the position estimated by our method. The color transition from red to blue illustrates the error in our method’s position estimation. The error range is depicted on the right side of each subplot. Figure 6 displays the trajectory error of our algorithm across these 11 representative sequences. The assessment system initially aligns actual and estimated values by utilizing the timestamps of the poses. Subsequently, it computes the disparity between each pair of poses. The figure depicts the absolute trajectory error, mean error, median error, and RMSE in meters. It can be seen that our algorithm has better effects than ORB-SLAM3 in multiple sequences and demonstrates better robustness.

### 6.2. 3D Reconstruction Experiments

**Figure 7:**
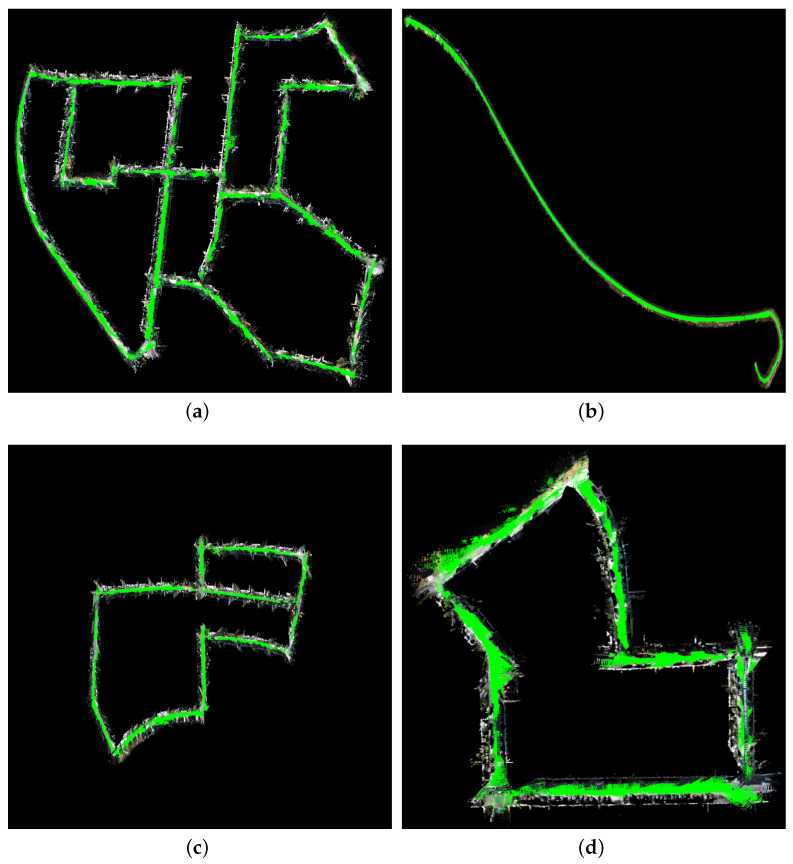

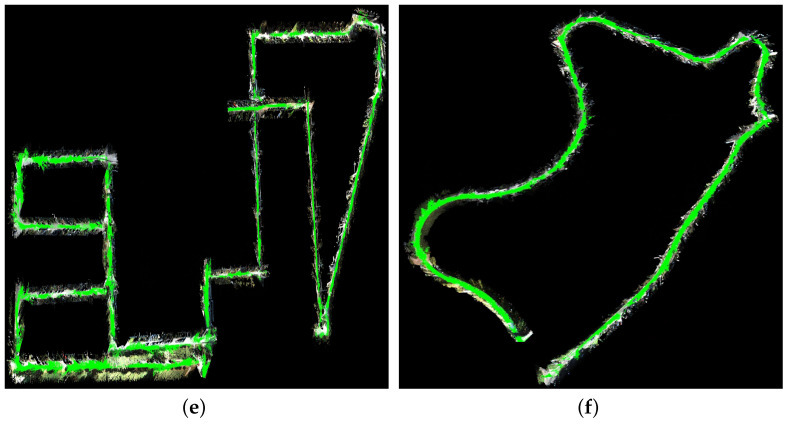
3D reconstruction based on road constraints, where green represents the road. (**a**) 00. (**b**) 01. (**c**) 05. (**d**) 07. (**e**) 08. (**f**) 09.

**Figure 8:**
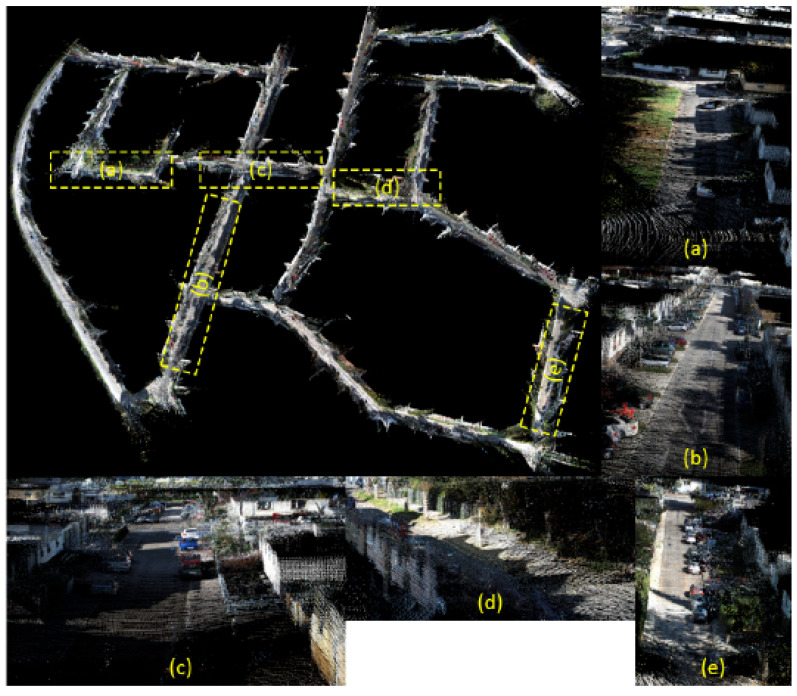
High-definition display of point clouds for some streets in the 00 sequence.The image in the top left corner is a 3D reconstruction of the entire city, and the other images depict details of its streets (**a**–**e**).

We conducted a 3D reconstruction experiment, as shown in Figure 7, which displays the overall point cloud of the corresponding dataset. The detected ground information is marked in green. According to Figure 5, the scene has a maximum size of 2000 m by 1200 m. As shown in Figure 8, we present a high-definition point cloud of a section of the street from sequence 00, where the clarity of the point cloud depends on the accuracy of the estimated trajectory. Thanks to the accuracy of the road surface extraction method [8] and the contribution of tensor voting [9] in the segmentation of spatial point clouds, the variance optimization constructed in our system has led to higher trajectory accuracy. It can be observed that we maintained significant clarity in a large-scale 3D reconstruction scene, demonstrating the high accuracy of our method.

## 7. Conclusions and Future Work

This paper proposes a trajectory estimation and mapping method based on ground information, with a focus on urban and flat road environments. The method integrates stereo vision and LiDAR data. Compared to the standalone LiDAR or vision methods, we achieve higher trajectory estimation accuracy. The method demonstrates stable performance in complex environments, achieving decimeter-level localization accuracy even at the urban scale while also enabling clear 3D reconstruction.

To leverage the advantages of stereo cameras and LiDAR while minimizing the computational costs, we align the ground information between the LiDAR map and visual images. Geometric features are extracted from the point cloud map and images through tensor voting to establish coplanarity constraints. Pose optimization is achieved through graph optimization and local window optimization.

In our experimental evaluation, we assessed the performance of our method in the urban environment of the KITTI dataset. The results indicate that our method outperforms the current state-of-the-art systems in both robustness and accuracy, showcasing a significant advantage in this particular setting. Future work will focus on optimizing the inference speed, exploring better integration of RGB and point-wise features, and incorporating additional operations on point clouds to further enhance our detection framework. Additionally, we will conduct further research on off-road scenarios, such as open areas with few features and bumpy terrains. 

## Figures and Tables

**Table 1:** Results of metrics’ absolute trajectory error (ATE) [M].

Sequence	ORB-SLAM3	Fast-LOAM	LeGO-LOAM	LOAM	Ours
00	**0.22**	0.96	5.03	0.96	0.26
01	2.09	2.80	21.02	2.68	**1.52**
02	10.82	1.57	2.80	5.04	**0.59**
03	0.43	1.09	1.32	1.16	**0.09**
04	0.18	1.43	1.63	1.39	**0.04**
05	0.38	0.80	0.93	0.71	**0.18**
06	0.36	0.72	0.84	0.72	**0.23**
07	0.39	0.55	0.77	0.54	**0.13**
08	2.87	**1.16**	1.58	1.18	1.93
09	0.88	1.29	1.48	1.21	**0.55**
10	1.27	1.77	1.86	1.61	**0.94**

## Data Availability

The data that support the findings of this study are openly available in TUM at https://www.cvlibs.net/datasets/kitti (accessed on 5 June 2024).
